# Bioactive Molecules and Their Mechanisms of Action

**DOI:** 10.3390/molecules24203752

**Published:** 2019-10-18

**Authors:** Dongdong Wang, Elke Heiss, Karel Šmejkal, Atanas G. Atanasov

**Affiliations:** 1 The Second Affiliated Hospital of Guizhou University of Traditional Chinese Medicine, Fei Shan Jie 32, 550003 Guiyang, China; dongdong.wang@univie.ac.at; 2 Institute of Clinical Chemistry, University Hospital Zurich, University of Zurich, Wagistrasse 14, 8952 Schlieren, Switzerland; 3 Department of Pharmacognosy, University of Vienna, Althanstrasse 14, 1090 Vienna, Austria; 4 Department of Natural Drugs, Faculty of Pharmacy, University of Veterinary and Pharmaceutical Sciences Brno, Palackého 1946/1, 61242 Brno, Czech Republic; 5 Department of Molecular Biology, Institute of Genetics and Animal Breeding of the Polish Academy of Sciences, 05-552 Jastrzębiec, Poland; 6 Institute of Neurobiology, Bulgarian Academy of Sciences, 23 Acad. G. Bonchevstr., 1113 Sofia, Bulgaria

Chronic inflammation with a wide spectrum of connected diseases (e.g., cancer, cardiovascular diseases (CVDs), diabetes, and neurodegenerative diseases) is the most significant cause of death in the world. Intense efforts to identify or better characterize compounds with anti-inflammatory potential are ongoing worldwide [1,2,3]. Traditional Chinese Medicine (TCM) has been practiced over centuries and has accumulated tremendous empirical knowledge on the treatment of such diseases. Nöst et al. identified several constituents from Huangqi Jianzhong Tang, a famous TCM herbal formula, which affected the secretion of pro-inflammatory cytokines in lipopolysaccharide (LPS)-stimulated U937 cells by combining in vitro assays to determine production of cytokines and LC-MS metabolomic techniques [4]. Gout is a chronic inflammatory disease evoked by the deposition of crystals of monosodium urate into joint tissues [5]. Lee et al. reported that epigallocatechin-3-gallate, an active component of green tea, prevented gouty inflammation by suppressing NLRP3 (NOD-, LRR-, and pyrin domain-containing protein 3) inflammasome activation via the blockade of mitochondrial DNA synthesis [6]. Xanthine oxidase (XO) catalyzes the conversion of hypoxanthine to xanthine and, in a second step, to uric acid, and represents a pivotal enzyme in gout and related disorders [7]. Hou et al. found that butein inhibited XO by occupying the catalytic center of XO to avoid the entrance of xanthine [8]. Furthermore, an improved method for its synthesis was described. A product obtained from an insect source *Holotrichia diomphalia* larvae possesses many pharmacological properties, including anti-inflammatory activity [9]. The study of Hong et al. indicated that the ethanol extract of *H. diomphalia* larvae inhibited the GATA-binding protein 3 (GATA-3)/Th2 signaling pathway and further exerted anti-asthmatic effects in a mouse model [10]. Curcumin represents one of the best studied natural compounds, with a range of reported bioactivities, including an anti-inflammatory effect [11,12,13]. The application of bibliometric analysis, which enables total-scale evaluation of specific research areas [14], allowed Yeung et al. to identify and quantitatively analyze 18,036 scientific publications on curcumin, outlining prevailing themes of the curcumin research field [15]. Although curcumin exhibits various benefits, it suffers from low bioavailability and poor solubility, which so far has limited its medicinal and therapeutic potential [16]. Hisamuddin et al. reported the anti-edematogenic and anti-granuloma activities of a synthetic, highly water-soluble curcuminoid analog, 5-(3,4-dihydroxyphenyl)-3-hydroxy-1-(2-hydroxyphenyl)penta-2,4-dien-1-one, using several animal models of inflammation [17].

Cancer is the second leading cause of death, and accounted for an estimated 9.6 million deaths worldwide in 2018 (https://www.who.int/news-room/fact-sheets/detail/cancer). Many small chemical entities are already used clinically for conventional and targeted cancer therapy, and much more recent research studies focus on the dissection of novel antitumor effects of diverse molecules [18,19,20]. As shown in this special issue, Mrkvová et al. performed high throughput screening of 2448 compounds for activating tumor suppressor p53 in the tumor cell line A375 [21]. The authors found that benzimidazoles activated p53 by downregulating Mdm2 and MdmX, which could be promising in the treatment of cancers overexpressing negative regulators of p53 [21]. Proinflammatory M1 macrophages promote tumoricidal response and suppress tumor growth, whereas M2(-like) macrophages contribute to a tumor environment by pampering cancer cell proliferation and survival [22]. Regarding macrophage polarization, Wei et al. reported that a polysaccharide isolated from *Radix Astragali* shifted macrophage phenotype polarization to M1 via the notch signaling pathway to further incite cytotoxic activity of macrophages against cancer cells, as well as decreased tumor volume and tumor weight in vivo [23]. In addition, Ye et al. found that 4β-hydroxywithanolide E from *Physalis peruviana* exhibited anti-colorectal cancer activity in vitro and in vivo by attenuating the wingless-type MMTV integration site family (Wnt)/β-catenin signaling pathway [24]. Moreover, the study of Luo et al. showed that vicenin II, a flavonoid glycoside (extracted also from *Dendrobium officinale*), inhibited transforming growth factor (TGF)-β1-induced epithelial–mesenchymal transition in lung adenocarcinoma cells via the deactivation of TGF-β/Smad and phosphoinositide 3-kinases (PI3K)/protein kinase B (Akt)/mammalian target of rapamycin (mTOR) signaling pathways, showing potential anti-metastatic activity [25]. Finally, Sperlich et al. discovered that pseudopterosin, produced by the sea whip of the genus *Antillogorgia*, inhibited proliferation and 3D invasion in triple-negative breast cancer cells by agonizing glucocorticoid receptor-α, as well as blocked elevation of levels of cytokines in a co-culture of peripheral blood mononuclear cells and MDA-MB-231 cells [26].

CVDs are disorders of the heart and blood vessels many times connected with chronic inflammation (https://www.who.int/health-topics/cardiovascular-diseases/). A total of 17.9 million people die each year from CVDs, an estimated 31% of all deaths worldwide (https://www.who.int/health-topics/cardiovascular-diseases/). Hypertension is a critical risk factor for the development of other CVDs and their complications. The study of Kim et al. indicated that *Citrus unshiu* peel contained both the contractile agonist synephrine and the anti-contractile substance nobiletin, which led to the abnormal vasoconstriction pattern exhibited by this herb [27].

Diabetes is a chronic metabolic disorder, which leads over time to serious hyperglycemia-induced damage to the heart, blood vessels, eyes, kidneys, and nerves (https://www.who.int/health-topics/diabetes). The most common type is type 2 diabetes, which occurs when the body becomes resistant to or possesses a relative lack of insulin (https://www.who.int/health-topics/diabetes). As currently used anti-diabetic drugs have marked side effects, continuous efforts to identify and characterize new molecules with anti-diabetic action are ongoing [28,29]. In this special issue, Yang et al. reported that alkali extract from *Amillariella mellea* fruiting body, polysaccharide-enriched fraction, lowered fasting blood glucose and improved glucose intolerance and insulin resistance in type 2 diabetic rats [30].

Neurodegenerative diseases such as Alzheimer’s disease, Parkinson’s disease, and multiple sclerosis are characterized by neuronal cell death, caused in part by an inflammatory process, mitochondrial alterations, and an elevation of oxidative stress [31,32]. Namsi et al. reported that an endogenous substance octadecaneuropeptide (ODN) induced N2a cell differentiation by the activation of the protein kinase A (PKA)/phospholipase C (PLC)/protein kinase C (PKC)/mitogen-activated protein kinase kinase (MEK)/extracellular signal-regulated kinase (ERK)-dependent signaling pathway, suggesting that ODN is a potent neuroprotective agent [32].

Osteoporosis is a highly prevalent bone disease caused by an inappropriate rate of bone resorption compared with bone formation, and the characterization of novel compounds with anti-osteoporotic potential is of high interest [33,34,35,36]. Yodthong et al. reported that L-quebrachitol promoted the proliferation, differentiation, and mineralization of pre-osteoblastic MC3T3-E1 cells by triggering the bone morphogenetic protein-2 (BMP-2)-response, as well as the runt-related transcription factor-2 (Runx2), mitogen-activated protein kinase (MAPK), and Wnt/β-catenin signaling pathway [37]. In addition, tetrahydroxystilbene glucoside, a unique component of the bone-reinforcing herbal preparation *Radix Polygoni Multiflori*, was shown to activate the PI3K/Akt pathway, and thus promote MC3T3-E1 cell proliferation and differentiation. Together with the proven influence on the expression of osteoprotegerin (OPG)/nuclear factor-κB ligand (RANKL)/macrophage colony-stimulating factor (M-CSF), this suggests that this tetrahydroxystilbene glucoside might be a potential therapeutic agent for osteoporosis [38].

Antimicrobial and antifungal properties of constituents of medicinal plants have been widely reported [39]. In our issue, Xiao et al. reported that berberine hydrochloride exhibited inhibitory effects on the growth of *Trichophyton mentagrophytes*, which might be associated with down-regulation of 14α-demethylase [40].

This special issue also includes two reviews. One summarized the available ethnobotanical, phytochemical, and pharmacological information about the traditional Amazonian medicinal tree *Maytenus macrocarpa* (Celastraceae), which is traditionally used mainly to treat rheumatism and, to a lesser extent, to heal wounds and to combat bronchitis and diarrhea [41]. The other one provided an overview of recent advances in the production of hybrid nanogels containing different biomolecules for various biomedical applications, and commented, among others, on their key physical consequences, functionalization, and advantages [42].

Overall, the Special Issue “*Bioactive Molecules and Their Mechanisms of Action*” gives a current snapshot on the multifaceted interdisciplinary character of this research topic, as evident in the wide range of addressed questions and employed methodologies. Considering the challenges in the field of drug discovery, this issue complements not only our knowledge on bioactives, but may also provide some novel ideas and inspiration for further investigation of diverse bioactive molecules with the potential for future therapeutic development.
